# *In Vitro* and *In Silico* Evaluation for the Inhibitory Action of *O. basilicum* Methanol Extract on *α*-Glucosidase and *α*-Amylase

**DOI:** 10.1155/2021/5515775

**Published:** 2021-07-06

**Authors:** Siba Shanak, Najlaa Bassalat, Raghad Albzoor, Sleman Kadan, Hilal Zaid

**Affiliations:** ^1^Faculty of Sciences, Arab American University, P.O Box 240, Jenin, State of Palestine; ^2^Faculty of Medicine, Arab American University, P.O Box 240, Jenin, State of Palestine; ^3^Qasemi Research Center, Al-Qasemi Academic College, P.O Box 124, Baqa-El-Gharbia 30100, Israel

## Abstract

Diabetes mellitus is a metabolic disease that predominates, nowadays. It causes hyperglycemia and consequently major health complications. Type II diabetes is the most common form and is a result of insulin resistance in the target tissues. To treat this disease, several mechanisms have been proposed. The most direct route is via inhibiting the intestinal enzymes, e.g., *α*-glucosidase and *α*-amylase, responsible for intestinal polysaccharide digestion that therefore would reduce the absorption of monosugars through the intestinal walls. In this study, we shed the light on this route by testing the inhibitory effect of *Ocimum basilicum* extract on the enzymes *α*-glucosidase and *α*-amylase *in vitro* and *in silico*. Experimental procedures were performed to test the effect of the *O. basilicum* methanol extract from aerial parts followed by the *in silico* docking. 500 *μ*g/mL of the extract led to 70.2% ± 8.6 and 25.4% ± 3.3 inhibition on *α*-glucosidase and *α*-amylase activity, respectively. Similarly, the effect of caffeic acid, a major extract ingredient, was also tested, and it caused 42.7% ± 3.0 and 47.1% ± 4.0 inhibition for *α*-amylase and *α*-glucosidase, respectively. Docking experiments were performed to predict the phytochemicals responsible for this robust inhibitory activity in the *O. basilicum* extracts. Several compounds have shown variable levels of inhibition, e.g., caffeic acid, pyroglutamic acid, and uvasol. The results indicated that *O. basilicum* can be a potent antidiabetic drug.

## 1. Introduction

Diabetes mellitus (DM) is a metabolic disorder. It results from the resistance to insulin or the reduced secretion of insulin. A major consequence of this disorder is the distorted carbohydrate, fat, and protein metabolism and the increased levels of serum glucose. This would result in hyperglycemia and elevated plasma LDL [1], which would cause damage in blood vessels and consequently microvascular and macrovascular disorders, including atherosclerosis, retinopathy, and nephropathy. Weight loss, polyphagia, blurred vision, polyuria, and polydipsia are additional complications [2]. These complications are the major causes of mortality in patients with diabetes [3]. Two major forms of diabetes predominate. In type I diabetes, which has a low prevalence, the pancreatic *β*-cells are labeled for destruction by the immune system, and insulin levels are distorted [4, 5]. The more common form, type II diabetes mellitus (T2DM), mainly results from insulin resistance in the target tissues [6]. A major consequence of this insulin disorder is an imbalance in carbohydrate, fat, and protein metabolism. Fasting plasma glucose levels, the postprandial levels of plasma glucose, and hemoglobin A1C levels are elevated to ≥7 mM, ≥11 mM, and 6.5%, respectively [7]. Prolonged hyperglycemia leads to the destruction of blood vessels and destruction in the heart, eyes, kidneys, and nervous system [3].

To treat type I DM, several routes can be followed. These include insulin replacement therapy and the transplantation of pancreatic islets [8]. Type II DM can be treated with drugs that act via different modes of actions. Some drugs target hepatic enzymes involved in gluconeogenesis and glycogenolysis for inhibition. Other drugs induce an increased production and secretion of insulin in the *β*-pancreatic cells. Even more, some drugs act on enzymes of skeletal muscles and adipose tissues and stimulate the two organs to increase blood glucose uptake [9, 10]. Another route of action is via drugs that target the inhibition of lipolysis, where imbalanced lipolysis is known to induce the reduced secretion of insulin and consequently hyperglycemia. Furthermore, abnormal lipolysis can result in lipotoxicity, with the accumulation of toxic lipid metabolites (ceramide, diacylglycerol, and fatty acyl CoA) in the liver, muscle, adipose tissues, and pancreas [11]. Drugs that target this side effect can relieve the injury and reduce the inflammation [12]. Anti-inflammatory drugs can treat the complications of diabetes, including the inflammatory responses [13]. The route for glycemic control under a microscope in this work introduces the inhibition of intestinal enzymes responsible for intestinal polysaccharide digestion and consequently the absorption of absorbable sugar monomers through the intestinal walls [14]. Examples include the mammalian *α*-glucosidase and *α*-amylase competitive inhibitors [15]. Digestive enzymes in the small intestine hydrolyze complex polysaccharides and disaccharides into smaller fragments of monosaccharides. The inhibition of these enzymes directly prevents the escape of the resulting monosaccharides to the bloodstream and the consequent utilization by the liver, muscle, and fat tissues.

Several antidiabetic drugs are confined by their limited action and pharmacokinetic influence and have side effects, e.g., biguanides and sulfonylurea. Traditional medicine utilizes herbal-based remedies by about 80% of the world's population. Herbal-derived active compounds or chemically modified herbal phytochemicals are used to produce safer pharmaceutically active drugs [16, 17]. Indeed, many plant extracts have shown antidiabetic effects in *in vitro* experiments, in animal test models, and in clinical trials. [17].

*Ocimum basilicum* is among one of the antidiabetic herbs [18]. The genus *Ocimum* belongs to the family Lamiaceae [19]. A number of species of *Ocimum* are used to treat different types of diseases, mainly the species *Ocimum basilicum,* also known as sweet basil—an herbaceous, perennial plant used in traditional medicine and also an ornamental plant [20, 21]. A number of virulent metabolites exist in this species that have strong action against diseases [22, 23]. It has a wide range of pharmacological activities, much as the antimicrobial effect. This was seen against *Aspergillus ochraceus* [24] for extracts from the hairy root of *O. basilicum* against species such as *P. aeruginosa* strains, *A. rhizogenes*, *P. fluorescens*, *X. campestris*, and *E. carotovora* [25] and for the leaf extract against *E. coli* and *Staphylococcus aureus* [26]. The essential oil extract from *O. basilicum* leaves showed an insecticidal activity against larval stages of *Culex tritaeniorhynchus*, *Aedes albopictus*, and *Anophelessubpictus* [27, 28]. *O. basilicum* contains several active antioxidant compounds, e.g., polyphenoid rosmarinic acid, a derivative of cinnamic acid [29]. Extracts from *Ocimum basilicum* aerial parts have robust anti-inflammatory activity against macrophages and human primary chondrocytes [30]. Extracts of *Ocimum basilicum* aerial parts have also shown antiplatelet activity through inhibiting ADP-induced platelet aggregation [31], anticonvulsant activity [32], and antithrombotic activity [33]. *O. basilicum* extract significantly showed antihyperlipidemic effects and could lower both plasma triglycerides (TG) and cholesterol in rats [31]. *Ocimum basilicum* aerial extracts (methanol, hexane, and dichloromethane) reported recently to augment glucose transporter-4 (GLUT4) translocation to the muscle plasma membrane in vitro and thus enhance glucose uptake [18].

*α*-Glucosidase (*α*-glucosidase, EC 3.2.1.20) is a carbohydrate hydrolase that breaks down terminal nonreducing (alpha-1 ⟶ 4)-linkage to release *α*-glucose residues. Two families of *α*-glucosidase are examined so far according to the primary structure [34]. The gene coding for human lysosomal *α*-glucosidase is about 20 kb long, and the structure for the protein it codes for has been resolved [35]. The Trp-516 and Asp-518 residues are crucial for the enzyme's catalytic functionality [36]. It was found that the conformation of the enzyme's active site is less stable than the whole enzyme conformation [37]. Deficiency in *α*-glucosidase may result in several disorders, e.g., Pompe disease or glycogen storage disease type II [38].

*α*-Amylase (*α*-amylase, EC 3.2.1.1) is an enzyme that hydrolyses alpha bonds of polysaccharides, such as starch and glycogen, to produce glucose and maltose. At least, two major forms of amylase are found in humans. Salivary *α*-amylase breaks starch into maltose and dextrin. The pancreatic *α*-amylase digests sugars in the diet to small saccharides such as maltose for the uptake in the small intestine on reaching the duodenum. Pancreatic *α*-amylase was discovered to bind with N-linked oligosaccharides of glycoproteins, which regulates the activities of glycoproteins related to the blood glucose level [39, 40]. Three *α*-amylase genes, AMY1 (the salivary *α*-amylase gene), AMY2A, and AMY2B (pancreatic *α*-amylase genes), form a cluster on chromosome 1 *P*21, with a pairwise sequence homology of 93%–94% [41, 42].

A number of distinct protein domains make up *α*-amylases: The catalytic domain has an eight-stranded alpha/beta barrel containing the active site, an ∼70-amino acid calcium-binding domain protrudes in the catalytic domain between alpha helix 3 and beta strand 3, and a Greek key beta-barrel domain is the carboxyl terminal. [43–46]. Acarbose is a complex oligosaccharide that serves as an oral inhibitor of *α*-glucosidase and *α*-amylase in the treatment of T2DM [47].

The progress in drug discovery has bloomed hugely nowadays. Chemoinformatics is used to scan hundreds of plausible protein ligands. One method for screening is the ligand-based approach. With the escalating number of resolved crystal structures, modeling for the binding of ligands to the target proteins can be configured. Docking is a structure-based method that acts at the atomic resolution for the screening of robust ligand-protein interactions, including the calculation for ligand position, orientation, inhibition constants, and binding affinities. In this study, we examined the inhibitory effect of methanol *O. basilicum* extract on *α*-glucosidase and *α*-amylase *in vitro* and *in silico*.

## 2. Materials and Methods

### 2.1. Preparation of Plant Material

The selected plants were extracted according to Kadan et al. [18] with methanol. 10 g of grounded OB was packed in the thimble of the Soxhlet apparatus and were extracted with 150 mL of methanol (MeOH) and then refluxed for 24 h to give a dark green extract. The yield of the extract was 1.05 g (10.5%). Supernatants obtained from the extract were passed through a 0.2 *μ*m filter and stored in aliquots at −80°C for further experimental work.

### 2.2. *α*-Amylase Inhibitory Method

The pancreatic *α*-amylase inhibition assay was performed according to Adisakwattana et al. [48] using acarbose as a positive control. Porcine pancreatic *α*-amylase (4 units/mL) was dissolved in 0.1 M sodium phosphate buffer, pH 6.9.

The plant extract, caffeic acid, or acarbose as a positive control of different dilutions was preincubated with 250 *μ*L of the enzyme solution at 37°C for 10 min. The reaction was initiated by adding 500 *μ*L of the substrate solution (1% starch in 0.1 M sodium phosphate buffer, pH 6.9). After 5 min of incubation, the reaction was stopped by adding 1 mL of 96 mM 3,5-dinitrosalicylic acid solution to the reaction mixture. The mixtures were heated at 100°C for 10 min in order to stop the reaction and then cooled to room temperature in a cold water bath. Subsequently, the reaction mixtures were diluted 10 times with distilled water. The absorbance was recorded at 540 nm using a spectrophotometer. The *α*-amylase inhibitory potential was calculated utilizing the following equation:

(1) $$\begin{array}{c} I\left(\%\right)=\frac{\left[{\text{ABS}}_{\text{blank}}-{\text{ABS}}_{\text{test}}\right]}{\left[{\text{ABS}}_{\text{blank}}\right]}*100\%, \end{array}$$

where *I* (%) is the *α*-amylase inhibitory percentage.

### 2.3. Intestinal *α*-Glucosidase Inhibitory Method

The assessment of intestinal *α*-glucosidase inhibitory activity was performed according to Kim et al. [49], with a slight modification. The reaction mixture consisting of plant extract, caffeic acid, or acarbose as a positive control at varying concentrations was premixed with 100 *μ*L of 0.1 M sodium phosphate buffer, pH 6.9. 15 *μ*L of *α*-glucosidase (0.1 unit/*μ*L) was added and preincubated at 37°C for 10 min. The reaction mixture was set to 750 *μ*L with distilled water. The reaction was initiated by adding 250 *μ*L of 20 mM p-nitrophenyl *α*-D-glucopyranoside and further incubated for 10 min. The reaction was terminated by the addition of 100 *μ*L of 0.1 M Na_2_CO_3_. The amount of released product (p-nitrophenol) was measured at 405 nm using a spectrometer.

The intestinal *α*-glucosidase enzyme inhibitory potential was measured utilizing the following equation:

(2) $$\begin{array}{c} I\left(\%\right)=\frac{\left[{\text{ABS}}_{\text{blank}}-{\text{ABS}}_{\text{test}}\right]}{\left[{\text{ABS}}_{\text{blank}}\right]}*100\%, \end{array}$$

where *I* (%) is the *α*-glucosidase inhibitory percentage.

### 2.4. Docking Experiments

Input PDB files were prepared for the natural compounds that were extracted from *O. basilicum*, including caffeic acid. The SMILES structures of the compounds were retrieved from the systemic IUPAC structures [50] and then converted to the PDB form using the Open Babel server [51]. These compounds were docked against the *apo* forms for the structures of the *α*-glucosidase enzyme (PDB: 5KZW) and the *α*-amylase enzyme (PDB: 1C8Q) with the AutoDock program, version 4.2 [52]. In each docking experiment, the receptor protein was kept rigid. Protein polar hydrogen atoms were added, and the input files were prepared using AutoDock tools [52]. Docking was performed within parallel rectangular boxes of 126 × 126 × 126 Å dimensions. The center of the grid was placed at the center of the mass of the original protein receptor in its *apo* form in crystal structures. A total of 20 independent docking runs were carried out for each compound against each of the enzymes starting from random positions. The PDB files were extracted and evaluated for the best-ranked fit of the enzyme-ligand interaction for each of the ligands.

## 3. Results

We have recently reported the phytochemical analysis of *O. basilicum* [18] with the objective to identify more potential antidiabetic active compounds (and test their potency in inhibiting carbohydrates digestive enzymes); the methanol *O. basilicum* extract from dried aerial parts was tested herein *in vitro* and *in situ*. The potential inhibitory effect of the extract on *α*-amylase and *α*-glucosidase was examined as described in the Materials and Methods section. Similarly, caffeic acid (as a major compound in the extract and one that displayed *in situ* inhibition) inhibitory effect was also tested.

**Figure 1:**
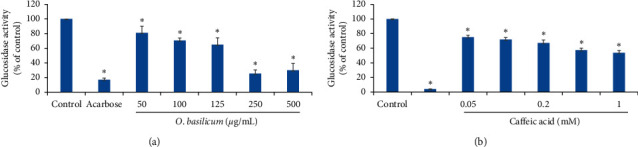
Effects of *O. basilicum* (a) and caffeic acid (b) on intestinal *α*-glucosidase. Values represent the mean ± SEM of three experiments. The *t*-test of statistical calculations was conducted using SPSS, version 23.0. ^*∗*^*p* < 0.05, which is considered significant as compared with controls.

The *in vitro* antidiabetic activities of *O. basilicum* methanol extract and for caffeic acid were investigated by the assessment of their pancreatic *α*-amylase and intestinal *α*-glucosidase inhibitory effects. Acarbose was used as a standard inhibitory drug. The results revealed that *O. basilicum* extract inhibited *α*-glucosidase in a dose-dependent manner and the IC_50_ value was 160 ± 10 *μ*g/mL (Figure 1(a) and Table 1). Caffeic acid also inhibited *α*-glucosidase in a dose-dependent manner, and the IC_50_ value was 1.05 ± 0.25 mM (Figure 1(b) and Table 1).

**Figure 2:**
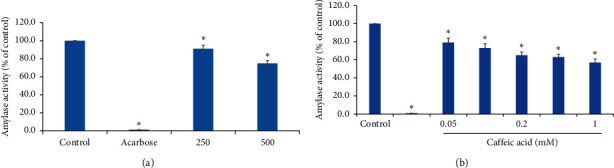
Effects of *Ocimum basilicum* (a) and caffeic acid (b) on pancreatic *α*-amylase. Values represent the mean ± SEM of three experiments. The *t*-test of statistical calculations was conducted using SPSS, version 23.0. ^*∗*^*p* < 0.05, which is considered significant as compared with controls.

*O. basilicum* inhibited also *α*-amylase yet at less potency compared with its inhibition to *α*-glucosidase. 500 *μ*g/mL of *O. basilicum* extract inhibited *α*-amylase by only 25.4% ± 3.3 (Figure 2(a)). Caffeic acid inhibited *α*-amylase in a dose-dependent manner and led to 42.7% ± 3.0 inhibition at 1 mM (Figure 2(b)).

**Figure 3:**
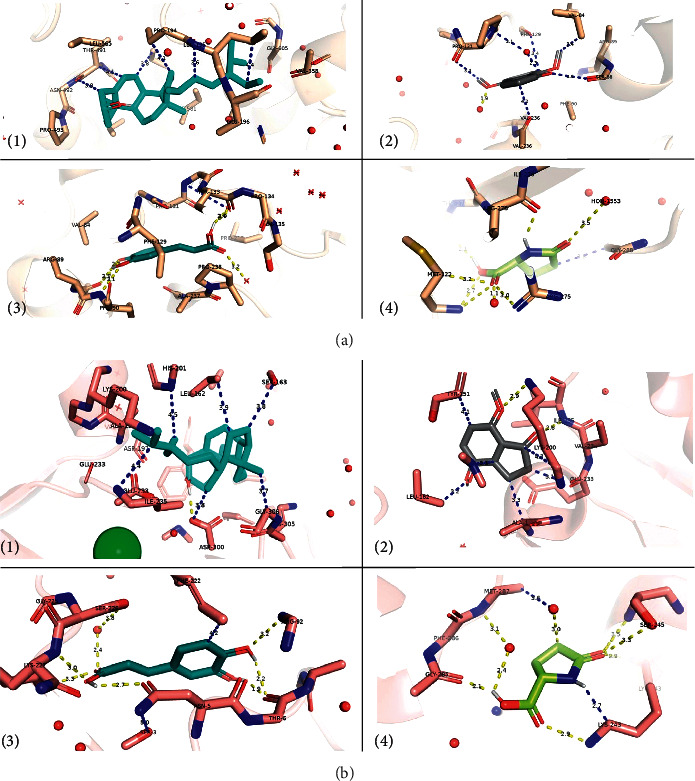
Binding interface between plausible inhibitors and (a) the *α*-glucosidase enzymes: (1) beta-sitosterol, (2) 4,7-dimethoxyindan-1-one, (3) caffeic acid, and (4) pyroglutamic acid; and (b) the *α*-amylase enzymes: (1) beta-sitosterol, (2) 4,7-dimethoxyindan-1-one, (3) caffeic acid, and (4) pyroglutamic acid. All amino acids that are within 5 Ǻ from the ligand are shown as sticks. The rest of the protein is shown in an 80% transparent cartoon model. Polar contacts are shown in yellow, whereas other possible contacts are in blue. The green ball in label (1) of subpart 3(b) refers to a chloride ion near the active site.

Results for the docking experiments met well with the *in vitro* evaluation. Phytochemicals screened in the extract from OB were tested for their binding affinities, inhibition constants, and the root mean square deviation (RMSD) values for the ligand structure upon docking from the reference one. Three compounds showed potent inhibition to both *α*-glucosidase and *α*-amylase: beta-sitosterol followed by 4,7-dimethoxyindan-1-one and then caffeic acid (Tables 2 and 3). Weak nonpolar contacts accounted for most of the interactions at the binding interface, with few polar contacts seen as hydrogen bonding between the ligand and the indicated amino acid residues (Figure 3).

**Figure 4:**
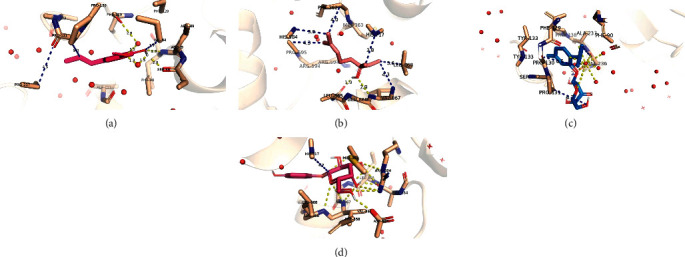
Binding interface between plausible inhibitors and the *α*-glucosidase enzyme: (a) E-isoeugenol, (b) linalool, (c) beta-D-glucopyranoside,5-methyl-2-1-methylethyl-phenyl, and (d) uvasol. All amino acids that are within 5 Ǻ from the ligand are shown as sticks. The rest of the protein is shown in an 80% transparent cartoon model. Polar contacts are shown in yellow, whereas other possible contacts are in blue.

Other potent inhibitors also existed that showed only an inhibitory effect against *α*-glucosidase but not *α*-amylase: E-isoeugenol (*K*_*i*_ = 22.33 *μ*M) followed by linalool (*K*_*i*_ = 78.91 *μ*M), beta-D-glucopyranoside,5-methyl-2-1-methylethyl-phenyl (*K*_i_ = 86.74 *μ*M), and finally uvasol (*K*_*i*_ = 118.93 *μ*M) in a descending order with respect to their inhibition constants (Figure 4). This might explain why the extract had more potent effect on the enzyme *α*-glucosidase than the *α*-amylase enzyme in the *in vitro* experiments. Indeed, the inhibition constants for the common inhibitory phytochemicals had very similar effects.

Pyroglutamic acid existed in both inhibitor lists for *α*-glucosidase and *α*-amylase, but the inhibition was predicted to be weak when compared with other phytochemicals (*K*_*i*_ = 298.88 *μ*M, 276.50 *μ*M for *α*-glucosidase and *α*-amylase, respectively, see Figure 3).

## 4. Discussion

*O. basilicum* has been reported as a potential antidiabetic herb, yet the action mechanisms and the potential antidiabetic compounds in *O. basilicum* that inhibit intestinal digestive enzymes were not discussed and some were not identified. Here, *O. basilicum* methanol extract and one of the major compounds, i.e., caffeic acid inhibited *α*-amylase and *α*-glucosidase in a dose-dependent manner. Docking experiments were undertaken to understand the mechanism by which the *O. basilicum* extracts would inhibit the two enzymes. Phytochemicals in the *O. basilicum* extract were screened for their inhibitory potency, binding interface, and structural fluctuations. The number of inhibitors that were predicted to work against *α*-glucosidase was twofold more than those inhibiting *α*-amylase (8 active phytochemicals for *α*-glucosidase vs 4 active phytochemicals for *α*-amylase). Still, the inhibition level of the four common phytochemicals was comparable for the two enzymes. This would justify the higher inhibition levels for *α*-glucosidase when compared with *α*-amylase *in vitro*. The deviations from the reference structure (the RMSD values) were highly reasonable for all plausible inhibitors. Of the four common inhibitors, the binding interface for caffeic acid and pyroglutamic acid showed more polar contacts than beta-sitosterol and 4,7-dimethoxyindan-1-one. Thus, more polarity did not contribute to a more stable binding interface and the binding free energy as the hydrophobic contacts did. On the other hand, the four inhibitors unique to *α*-glucosidase (E isoeugenol, linalool, beta-D-glucopyranoside,5-methyl-2-1-methylethyl-phenyl, and uvasol) showed a good hydrophilic interface with the surrounding amino acid residues and the coordinating water molecules.

Previous works suggested that glucosidase inhibitors and amylase inhibitors are a class of compounds that help controlling diabetes by diminishing the absorption of glucose from the intestine [53]. Some research also suggested that the water/methanol extract of food materials displayed the antidiabetic activity against amylase and glucosidase [54, 55]. These results provide intense rationale for further *in vivo* study and drug identification.

## Figures and Tables

**Table 1:** The IC_50_ values of *O. basilicum* and caffeic acid for intestinal *α*-glucosidase and *α*-amylase.

	*O. basilicum*	Caffeic acid
*α*-Glucosidase	160 ± 10 *μ*g/mL	1.05 ± 0.25 mM
*α*-Amylase	>500 *μ*g/mL	>1 mM

**Table 2:** Binding free energies, inhibition constants, and the RMSD values calculated by AutoDock for ligand binding to *α*-glucosidase.

OB phytochemical	AutoDock binding free energy (kcal/mol)	AutoDock inhibition constant (*K*_*i*_)	RMSD for the ligand from the reference structure (Å)
**2-Hydroxymethyl-6-3,4,5-trihydroxy-2-hydroxymethyl**	−3.37	3.36 mM	11.463
**3,3-Dihydroxyacrylic acid**	−3.33	3.61 mM	15.571
**4,7-Dimethoxyindan-1-one**	−5.79	56.88 *μ*M	8.094
**Alpha-hydroxyhydrocaffeic acid**	−4.29	721.41 *μ*M	7.700
**Alpha-linolenic acid**	−4.74	337.32 *μ*M	9.710
**Arabinitol**	−1.35	102.52 mM	49.393
**Beta-D-galactofuranose**	−4.19	853.24 *μ*M	6.313
**Beta-D-glucopyranoside,5-methyl-2-1-methylethyl-phenyl**	−5.54	86.74 *μ*M	12.272
**Beta-D-ribofuranose**	−3.64	2.16 mM	26.731
**Beta-sitosterol**	−7.93	1.53 *μ*M	30.109
**Caffeic acid**	−5.45	100.99 *μ*M	10.935
**Cyanuric acid**	−4.66	382.74 *μ*M	14.773
**D-Xylopyranose**	−3.88	1.42 mM	28.285
**D-Xylose**	−2.26	22.04 mM	23.337
**E-but-2-ene-1-4-diol**	−3.79	1.67 mM	9.428
**E-Isoeugenol**	−6.35	22.33 *μ*M	10.075
**Glucopyranose**	−2.97	6.70 mM	6.426
**Hydroquinone**	−4.55	461.18 *μ*M	8.566
**Inositol**	−4.48	520.07 *μ*M	38.863
**Linalool**	−5.60	78.91 *μ*M	48.561
**Linoleic acid**	−3.46	2.91 mM	17.302
**Mannitol**	−3.26	4.05 mM	9.698
**Palmitic acid**	−3.91	1.35 mM	59.142
**Pentane-1,2,5-triol**	−3.23	4.26 mM	49.447
**Pyroglutamic acid**	−4.81	298.88 *μ*M	16.630
**Talose**	−2.91	7.34 mM	6.730
**Uvasol**	−5.35	118.93 *μ*M	45.174
**Glycerol**	−1.11	152.58 mM	—
**L-Valine**	−2.93	7.10 mM	—
**Succinate**	−3.77	1.71 mM	—
**Threitol**	−1.58	69.02 mM	—
**Urea**	−2.96	6.82 mM	—

**Table 3:** Binding free energies, inhibition constants, and the RMSD values calculated by AutoDock for ligand binding to *α*-amylase.

OB phytochemical	AutoDock binding free energy (kcal/mol)	AutoDock inhibition constant (*K*_*i*_)	RMSD for the ligand from the reference structure (Å)
**2-Hydroxymethyl-6-3,4,5-trihydroxy-2-hydroxymethyl**	−3.26	4.09 mM	45.065
**3,3-Dihydroxyacrylic acid**	−3.99	1.20 mM	56.124
**4,7-Dimethoxyindan-1-one**	−5.77	59.10 *μ*M	44.384
**Alpha-hydroxyhydrocaffeic acid**	−4.67	379.57 *μ*M	64.753
**Alpha-linolenic acid**	−4.03	1.11 mM	50.314
**Arabinitol**	−2.05	31.56 mM	41.922
**Beta-D-galactofuranose**	−3.52	2.61 mM	48.439
**Beta-D-glucopyranoside,5-methyl-2-1-methylethyl-phenyl**	−4.02	1.14 mM	44.414
**Beta-D-ribofuranose**	−3.35	3.48 mM	44.547
**Beta-sitosterol**	−8.38	719.79 nm	48.783
**Caffeic acid**	−5.25	140.92 *μ*M	68.360
**Cyanuric acid**	−4.79	310.58 *μ*M	81.408
**D-Xylopyranose**	−3.48	2.83 mM	45.736
**D-Xylose**	−2.48	15.29 mM	70.312
**E-but-2-ene-1-4-diol**	−4.30	708.76 *μ*M	39.448
**E-Isoeugenol**	−4.64	397.34 *μ*M	48.923
**Glucopyranose**	−3.80	1.65 mM	46.170
**Hydroquinone**	−4.13	933.22 *μ*M	44.919
**Inositol**	−3.78	1.68 mM	90.688
**Linalool**	−4.13	933.83 *μ*M	53.531
**Linoleic acid**	−3.75	1.77 mM	79.184
**Mannitol**	−2.41	17.08 mM	44.479
**Palmitic acid**	−4.02	1.13 mM	57.625
**Pentane-1,2,5-triol**	−2.99	6.45 mM	36.675
**Pyroglutamic acid**	−4.85	276.50 *μ*M	58.241
**Talose**	−2.43	16.69 mM	64.498
**Uvasol**	−4.39	603.30 *μ*M	45.065
**Glycerol**	−1.35	102.76 mM	—
**L-Valine**	−2.47	15.48 mM	—
**Succinate**	−4.00	1.16 mM	—
**Threitol**	−0.80	259.66 mM	—
**Urea**	−2.68	10.78 mM	—

## Data Availability

Data are available upon reasonable request to the corresponding author.
